# Changes and Determinants of Health-Related Quality of Life in Patients with Work-Related Traumatic Musculoskeletal Injuries: A Longitudinal Analysis of HRQoL and the EQ-VAS

**DOI:** 10.1007/s10926-025-10304-4

**Published:** 2025-06-23

**Authors:** Ilda Adrovic, Michaela Coenen, Stefan Simmel, Sandra Kus

**Affiliations:** 1 Institute for Medical Information Processing, Biometry, and Epidemiology–IBE, Chair of Public Health and Health Services Research, Faculty of Medicine, Ludwig-Maximilians-Universität in Munich, Elisabeth-Winterhalter-Weg 6, 81377 Munich, Germany; 2 Pettenkofer School of Public Health, Munich, Germany; 3 Department for Rehabilitation, BG Hospital Murnau, Murnau, Germany

**Keywords:** Polytrauma, Fracture, Occupational accident, Biopsychosocial, Quality of life

## Abstract

**Purpose:**

This study aims to analyze change over time in health-related quality of life and the current overall health status of patients with severe musculoskeletal injuries, specifically focusing on those who have undergone inpatient rehabilitation following work-related injuries.

**Methods:**

Data were sourced from the *icfPROreha* research project (DRKS-ID: DRKS00014857), involving a multicenter longitudinal study conducted across ten German clinics. The study population comprised patients who had sustained severe musculoskeletal work-related injuries. The EuroQol 5 Dimensions 5 Levels (EQ-5D-5L) was used to assess changes in health-related quality of life (HRQoL) measured at admission (t1), discharge (t2), and four follow-up periods up to 78 weeks after discharge. Descriptive analyses were conducted to illustrate the development of HRQoL across all time points. In addition, the current overall health status was measured using the EuroQol Visual Analogue Scale (EQ-VAS) at the same time points. Multilevel growth models were used to analyze change over time in EQ-VAS scores, considering the biopsychosocial perspective of health as indicated by the International Classification of Functioning, Disability, and Health (ICF), developed by the WHO.

**Results:**

In total, 698 patients [males: 75.2%; mean age 47.5 years (SD ± 12.3)] with severe musculoskeletal work-related injuries were included in the analyses. The mean EQ-VAS at baseline was 50.3 and the mean EQ-5D-5L index was 0.6. Descriptive analyses of the EQ-5D-5L showed significant improvements across all five dimensions of HRQoL after discharge, with the most pronounced changes observed in mobility and usual activities. Moreover, the study demonstrated a significant improvement in the EQ-VAS over time. Baseline EQ-VAS had a substantial influence on subsequent changes, with various factors such as psychological well-being, visible consequences and severity of injury affecting recovery outcomes. Notably, differences in health status over time were observed across different injury types.

**Conclusion:**

The study demonstrates significant enhancements in the current overall health status among patients with traumatic musculoskeletal injuries following workplace or commuting accidents during inpatient rehabilitation. Furthermore, these findings emphasize the application of the ICF framework in capturing the multidimensional aspects of patient recovery. Despite improvements, patients' health status did not reach the levels observed in the general population, indicating the need for ongoing support and targeted interventions to ensure long-term recovery.

**Supplementary Information:**

The online version contains supplementary material available at 10.1007/s10926-025-10304-4.

## Introduction

Work-related diseases and injuries have long been a significant global public health concern, contributing to substantial morbidity and mortality. In 2016, the World Health Organization (WHO) and the International Labour Organization (ILO) reported that work-related diseases and injuries were responsible for the deaths of 1.9 million people worldwide, with 360,000 deaths directly attributable to occupational injuries [1]. Beyond the human cost, these conditions also impose a significant economic burden on the healthcare system, including lost productivity, increased healthcare expenditures, and long-term disability compensation. Tompa et al. [2] estimated the economic burden of work injuries and diseases in five European countries. The researchers found that, in terms of gross domestic product (GDP), the total costs ranged between 2.7% (Finland) and 10.4% (Poland). For Germany, costs amounting to 3.3% of GDP are reported. As reported by Tompa et al., indirect costs (e.g., due to absenteeism and reduced workability, payroll, and fringe benefits) constitute the most substantial component of the economic burden, followed by direct costs and intangible costs. In Germany, the authors estimate the total annual economic burden of work injuries and diseases to be 107.129 million euros. These statistics underscore the need for effective preventive measures and post-injury rehabilitation.

In 2023, Germany recorded 783,426 workplace accidents, including 381 fatalities, and 184,355 commuting accidents, with 218 fatalities. Among these incidents, 13,965 resulted in either pension payments or death benefits, indicating the severe nature of these injuries. The risk of workplace accidents is significant, with a rate of 18.09 accidents per 1000 full-time equivalent employees [3]. Notably, over 30% of these accidents occur during movement, such as walking, while 20% are due to manual handling of objects, including securing, binding, and lifting tasks. The majority of fatalities result from falls, particularly from heights, and loss of control over transportation means [4]. These incidents often lead to severe musculoskeletal injuries, which can have lasting consequences for the affected individuals.

Musculoskeletal health is integral to the functionality of the human body, particularly the locomotor system, which comprises muscles, bones, joints, and surrounding connective tissues. Conditions affecting the musculoskeletal system are typically characterized by persistent pain and restrictions in movement and dexterity, significantly impairing an individual's ability to work and engage in daily activities. Common musculoskeletal disorders include low back pain, neck pain, rheumatoid arthritis, fractures, and amputations [5, 6]. Globally, these conditions are the leading contributors to chronic pain, physical functional impairments, and a diminished quality of life [7]. Consequently, musculoskeletal disorders represent the highest contributor to the demand for rehabilitation services worldwide [5].

Recent data from the 2023 TraumaRegister DGU provide further insight into the prevalence and severity of injuries in Germany. The register documented approximately 30,806 individuals with severe injuries, of which 3771 resulted in fatalities. Additionally, 4591 individuals suffered polytrauma, defined as significant injuries to at least two body regions, often accompanied by one or more physiological complications. Despite these alarming figures, the mortality rate following trauma has shown a continuous decline over the past decade, reaching approximately 7.9% in 2022 [8]. This improvement in survival rates has shifted the focus of medical care from merely ensuring survival to enhancing the quality of life for trauma survivors. Post-traumatic quality of life and the reestablishment of social participation have emerged as critical outcomes for evaluating the success of treatment during inpatient rehabilitation. Inpatient rehabilitation in Germany provides intensive, multidisciplinary care in specialized clinics to help patients recover from illness, injury, or surgery. Treatments like physiotherapy, occupational therapy, and psychological support aim to restore function and enable a quick, lasting return to daily life and work.

In Germany, the legal framework for work-related accidents is defined under Sect. 8, Paragraph 1 of the German Social Code Book VII (SGB VII), which covers accidents occurring to insured persons during professional duties or insured activities. Additionally, Sect. 8, Paragraph 2 of the German SGB VII includes those accidents occurring during the commute to or from the place of insured activity are also considered work accidents [9]. In the following of this paper, work-related accidents will be used to cover both types of incidents previously mentioned. Germany’s accident insurance system provides comprehensive coverage, including medical rehabilitation services, support for participation in working life and community activities, long-term care benefits, and financial compensation [10, 11]. Additionally, work-related accidents in Germany are treated in specialized clinics known as BG Hospitals, which provide care specifically for such incidents [12].

While the legal and medical frameworks provide essential support for individuals affected by work-related injuries, there is a growing awareness that embracing a more comprehensive and holistic approach to health is necessary. The traditional medical perspective often focuses on the physical aspects of health, but a biopsychosocial perspective, which considers the interaction between biological, psychological, and social factors, offers a more comprehensive understanding of health and well-being. The ICF, developed by the WHO, provides a valuable framework for this approach. The ICF incorporates multidimensional concepts: functioning, which includes body functions (e.g., pain), body structures (e.g., spine structures), activities (e.g., self-care), participation (e.g., social participation), and disability, which relates to impairments, activity limitations, or participation restrictions. Additionally, the ICF considers contextual factors, including environmental factors (e.g., social support) and personal factors (e.g., age) [13].

Despite the recognized importance of a holistic view, there remains a gap in scientific studies that systematically apply a biopsychosocial concept to assess the health-related quality of life (HRQoL) in patients, particularly over extended follow-up periods. HRQoL instruments concentrate on activities and participation, as these components are deemed most important to patients and society, and they are applicable to all health conditions and allow for the comparison of functioning and health across different health conditions, populations, and interventions [14]. Still, there is a gap in research that incorporates also the ICF framework in order to evaluate long-term outcomes and HRQoL in individuals, especially with severe musculoskeletal injuries.

The current study aims to analyze change over time in EQ-VAS in patients with severe musculoskeletal work-related injuries, from their admission to inpatient rehabilitation through a follow-up period of one and a half years after discharge from inpatient rehabilitation.

The specific research questions of this study are as follows:

1. How does HRQoL, measured by EQ-5D-5L, develop from admission to inpatient rehabilitation through 78 weeks after discharge?
2. How does current overall health status (measured via EQ-VAS) change over time during and after inpatient rehabilitation, as assessed using multilevel growth modeling?
3. Which baseline biopsychosocial factors are associated with individual differences in the trajectory of current overall health status over time, as examined using growth modeling?

## Methods

### Study Design

The data for this study were sourced from the *ICF-based prediction of outcomes in rehabilitation after trauma – icfPROreha* research project (www.icf-proreha.de). The project spanned four phases from April 2017 to October 2021. Phases 1 and 2 involved the preselection of potential predictors and the definition of measures to assess the potential predictors of return to work (RTW). Phase 3 comprised a multicenter longitudinal study with a follow-up of 18 months, while phase 4 aimed to develop rehabilitation recommendations and guidelines based on the study findings. The primary aim of icfPROreha was to predict RTW outcomes 78 weeks after discharge [16]. This project was a collaboration between the Chair of Public Health and Health Services Research (IBE) of the LMU Munich, the BG Rehabilitation Department of BG Unfallklinik Murnau, and the rehabilitation departments of nine other clinics across Germany.

The multicenter longitudinal study was conducted from August 2018 to August 2021 in the rehabilitation departments of the clinics mentioned above. Sociodemographic and injury-specific data were collected. Furthermore, the biopsychosocial perspective was addressed by collecting data on functioning and contextual factors, using a comprehensive ICF-based assessment tool [16]. Supplementary Table 5 provides an overview of questions used to address the biopsychosocial perspective.

The study protocol received approval from the Ethical Committee of the Medical Faculty at LMU Munich (Project Number: 18-329, approved on June 27, 2018) as well as from the Ethical Committees of the involved rehabilitation departments. Furthermore, the study was registered in the German Register of Clinical Studies (DRKS-ID: DRKS00014857). All participants provided written informed consent prior to their inclusion in the study.

### Study Population

Severely injured individuals admitted to participating clinics between August 2018 and December 2019 were included. Inclusion criteria were as follows: (1) age between 18 and 65 years; (2) diagnosed with severe musculoskeletal injuries, as per the German injury classification [17]; (3) commenced first inpatient rehabilitation within 16 weeks post-accident; (4) proficiency in spoken and written German; (5) a clear understanding of the study's objectives and procedures; and (6) provided informed consent. Individuals who met one of the following criteria were excluded from participation in the study: (1) significant nerve tract injuries, such as spinal injuries with neurological symptoms; and (2) cranio-cerebral injuries classified as SHT grade II [17]. For the current paper, only patients who had sustained work-related accidents were included.

### Assessment Tools

In Phases 1 and 2 of the icfPROreha project, experts developed a comprehensive assessment tool to capture functioning, contextual factors, injury-specific details, and sociodemographic data [16]. Standardized questionnaires were employed whenever possible to document potential predictors.

To measure HRQoL, this study employs the European Quality of Life instrument, EQ-5D-5L, which assesses five dimensions of health and includes a visual analogue scale (EQ-VAS) to provide a quantitative measure of an individual's current overall health status [18]. The five dimensions captured by the EQ-5D-5L questionnaire are mobility, self-care, usual activities, pain/discomfort, and anxiety/depression. Each dimension is rated on a 5-point Likert scale specifying five levels of severity: no (level 1), slight (level 2); moderate (level 3; severe (level 4); and extreme problems (level 5), allowing the description of 3125 health states [15, 18]. These possible unique health states can be linked to utilities. A collection of preference assessments from the general population concerning various health states was obtained and utilized to estimate EQ-5D Index scores [15, 19]. For the German normative population, the health states range from −0.205 (worst possible health) to 1 (best possible health), with one study indicating a mean EQ-5D Index value of 0.88 and a standard deviation of 0.18 [19]. As negative EQ-5D Index scores are possible, utilities can also represent a HRQoL worse than death. Additionally, the EQ-5D-5L includes a visual analogue scale (EQ-VAS) to assess current overall health status, which ranges from 0 (worst imaginable health state) to 100 (best imaginable health state). While the literature reports varying mean EQ-VAS values for the German population, in this specific mentioned study, the mean EQ-VAS is 71.6 with a standard deviation of 21.4 [19]. For the aim of this study, the EQ-VAS was used to analyze changes over time for patients with severe musculoskeletal injuries following inpatient trauma rehabilitation.

Other assessment tools included the WHO Disability Assessment Schedule (WHODAS) 2.0 for evaluating individual limitations and restrictions in activities and participation [20], the Patient Health Questionnaire (PHQ-4) for emotional functions [21], the General Self-Efficacy Short Scale (ASKU) for self-efficacy [22], the Resilience Scale (RS-13) for resilience [23], and the Alcohol Use Disorders Identification Test (AUDIT-C) for alcohol consumption and misuse [24].

### Data Collection and Follow-up

Patients who met the inclusion criteria and provided written informed consent were enrolled in the study. The assessment tool was completed at admission to the rehabilitation department (t1). HRQoL data were additionally recorded at discharge (t2) and at 12 weeks (t3), 26 weeks (t4), 52 weeks (t5), and 78 weeks (t6) after discharge. Assessments at t1 and t2 were conducted electronically using tablets, while data from t3 to t6 were collected either via telephone or written interviews. Data documentation was managed using the Research Electronic Data Capture (REDCap) system [25]. All follow-up data were collected and entered by trained interviewers.

### Statistical Analyses

Standardized questionnaires and instruments were analyzed according to their respective manuals and guidelines. Sociodemographic and injury-related variables were descriptively analyzed. The EQ-5D Index was generated for individual patients at different time points.

The dependent variable for the multilevel growth model was the current overall health status, measured by the EQ-VAS, ranging from 0 (worst imaginable health state) to 100 (best imaginable health state), assessed at six time points (t1 to t6). The independent variables (fixed effects) included a comprehensive set of baseline variables (measured at t1), selected based on previous literature and the ICF framework. These variables were considered in model 4a and 4b to explore their potential influence on the trajectory of EQ-VAS scores over time. In addition, descriptive variables were used to characterize the study population and are presented in Table 1. These include variables like sex, age, BMI, education level, employment status, and type of injury.

**Table 1:** Sociodemographic and injury-specific data at admission to first inpatient rehabilitation after trauma (t1) (*n* = 698), m = mean, SD = standard deviation

Sociodemographic and injury-specific variables	*N* (%)
Male	525 (75.2%)
Age at admission, m (SD)	47.5 (12.3)
BMI, m (SD)	27.7 (5.6)
Education	
No graduation	24 (3.4%)
Graduation up to grade 9	252 (36.1%)
Graduation up to grade 10	247 (35.4%)
Graduation up to grade 12	175 (25.1%)
Vocational education^*^	619 (88.7%)
Language: Cultural background^*^ – German	648 (92.8%)
Living situation	
Alone	190 (27.2%)
With others	508 (72.8%)
Children	
No children	431 (61.8%)
One or more	267 (38.2%)
Social status	
(a) Income (monthly household net income (€))	
Below 1700 €	132 (18.9%)
1700 €–2,300 €	150 (21.5%)
2300 €–3,200 €	143 (20.5%)
3200 € and more	185 (26.5%)
No information	88 (12.6%)
(b) Main Earner	
Yes	296 (42.4%)
No	248 (35.5%)
No information	154 (22.1%)
Employment status	
(a) Situation pre-accident	
Part-time employed	73 (10.5%)
Full-time employed	616 (88.3%)
Not working	9 (1.2%)
(b) Employment Type	
Self-employed	69 (9.9%)
Dependent employed	629 (90.1%)
Type of injury	
Extensive or deep injuries of the skin and soft tissue mantle; amputation injuries; muscle compression syndromes (compartment syndromes); thermal or chemical damage	41 (5.9%)
Injuries to the great vessels	5 (0.7%)
Severe chest or abdominal injuries with organ involvement including kidneys or urinary tract	47 (6.7%)
Complex fractures of the large tubular bones, especially multiple or open fractures	211 (30.2%)
Severe injuries to large joints	365 (52.3%)
Severe injuries to the hand	41 (5.9%)
Complex fractures of the facial skull and torso skeleton	180 (25.8%)
Multiple injuries with severe manifestations	41 (5.9%)
Treatment pre-admission to inpatient rehabilitation	
Physiotherapy	532 (76.2%)
Occupational therapy	176 (25.2%)
Massage/lymphatic drainage	340 (48.7%)
Medical training therapy	88 (12.6%)
Psychological therapy	46 (6.6%)
Other	56 (8.0%)
No treatment	120 (17.2%)
EQ-5D-5L at baseline	
EQ-5D Index, m (SD)	0.6 (0.3)
EQ-VAS, m (SD)	50.3 (20.9)

Medical training therapy: Medical training therapy or medical exercise therapy is part of the rehabilitation process that can be used to help patients recover. It helps to activate movement patterns, build muscular strength and endurance, improve coordination, and enhance overall physical performance

^*^Dichotomously coded variable (yes/no), only the result (*n* and %) is shown in relation to the “yes” response option

Given the hierarchical and clustered nature of the data, multilevel growth models with three levels were used to explore change over time in EQ-VAS over 78 weeks after discharge from the inpatient rehabilitation. For this, a vertical, long-form dataset was generated with all time points listed sequentially and sorted by patient ID’s [26]. The methodology outlined by Boscardin et al. [27] for growth modeling was selected for its ability to address missing data and to account for varying intervals between measurement points, thereby enabling the inclusion of participants with incomplete or irregular follow-up schedules. Moreover, growth modeling in a longitudinal study design follows the approach that data from at least three points are required. Hence, data from only a single time point or two were excluded from the present analyses, as the follow-up data for the EQ-5D-5L contained missing values.

Using the three-level growth model, time was nested within patients and patients were nested within clinics, with time as our level-1 variable, the patient IDs as our level-2 variable, and clinic as the level-3 variable. The EQ-VAS was the outcome variable. Initially, an unconditional model without predictors (Model 1) was estimated to assess variation in EQ-VAS between patients and between clinics. Additionally, the Intraclass Correlation Coefficient (ICC) was calculated to indicate how much of the total variation in EQ-VAS is accounted for by the patients and the clinics. For the ICC, the ratio of the intercept variance to total variance (intercepts + residual) needs to be calculated [26]. This first model addressed our first research question about the average EQ-VAS for patients and if it varied between them.

Subsequently, time was included as a fixed effect (Model 2), followed by the baseline EQ-VAS (Model 3), to examine the influences on change over time. The model fit was assessed using the Akaike Information Criterion (AIC) for each model [26, 28]. Known influential factors from the literature were added as fixed effects to control for our study population (Model 4a). Predisposing factors as age, gender, and education were found to have an influence on the current overall health status [29–32], but also enabling factors as income and living situation were included in the Model [30]. Furthermore, other studies have identified clinical factors (treatment, severity of injury, comorbidities, pain), psychological factors (self-efficacy, sleep, depression, subjective health status, life satisfaction before the accident), behavioral factors (smoking, alcohol use), and social factors (family support, family situation, professional support) as influential on HRQoL [29–33]. To examine potential effects of biopsychosocial aspects, additional variables related to functioning and contextual factors were included in the final Model 4b. This extension aimed to reflect a more holistic view of health, consistent with the ICF framework, by incorporating a broader range of biopsychosocial and environmental factors. Although the inclusion of these additional predictors served to better capture the complexity of recovery, the final model was selected based on model fit criteria. Stepwise variable selection, using the AIC, guided the final model choice. Variables with *p* values < 0.1 were examined in more detail and are highlighted in the tables, as such associations may indicate clinically relevant trends, even if they do not reach conventional levels of statistical significance. All statistical analyses were performed using R (Version 4.3.3, R Foundation for Statistical Computing, Vienna, Austria). Coding assistance and coding optimization were provided by OpenAI ChatGPT (Version GPT-4, OpenAI, San Francisco, CA, USA).

## Results

### Study Population

Out of 797 patients initially included from August 2018 to August 2021, 775 remained in the multicenter prospective study after excluding 22 patients. For the current study, 698 patients were considered as they met the additional criteria of a work-related accidents. Supplementary Fig. 1 illustrates the flowchart on inclusion and exclusion of the study cohort.

Table 1 presents the baseline characteristics of the study population. The cohort consisted of 525 males (75.2%) with an overall mean age of 47.5 years (SD ± 12.3). The mean EQ-VAS at baseline was 50.3, and the mean EQ-5D-5L index was 0.6. The majority (*n* = 365, 52.3%) had severe injuries to large joints, and a single localization of injury was noted in 502 patients (71.9%). On average, patients were admitted to inpatient rehabilitation 37.3 days after discharge from the acute care hospital. The mean duration of inpatient rehabilitation was 44.4 days, with a median duration of 35 days and a range of 11–520 days. Supplementary Fig. 2 illustrates the distribution across ten different work sectors, with the majority from production and manufacturing (19.9%) and construction (19.6%). Supplementary Table 1 provides a more detailed overview of baseline characteristics.

### Follow-up

The follow-up data for the EQ-5D had missing values at various time points: 3 at t2, 64 at t3, 61 at t4, 40 at t5, and 2 at t6. A total of 45 IDs were excluded from the analysis entirely due to the absence of data from two time points, while 22 IDs were excluded due to the absence of data from a single time point. This resulted in an analysis sample of 631 patients. The vertical, long-form dataset employed for growth modeling comprised 3491 observations.

### Descriptive Analyses: Change Over Time in HRQoL (EQ-5D-5L)

**Fig. 1:**
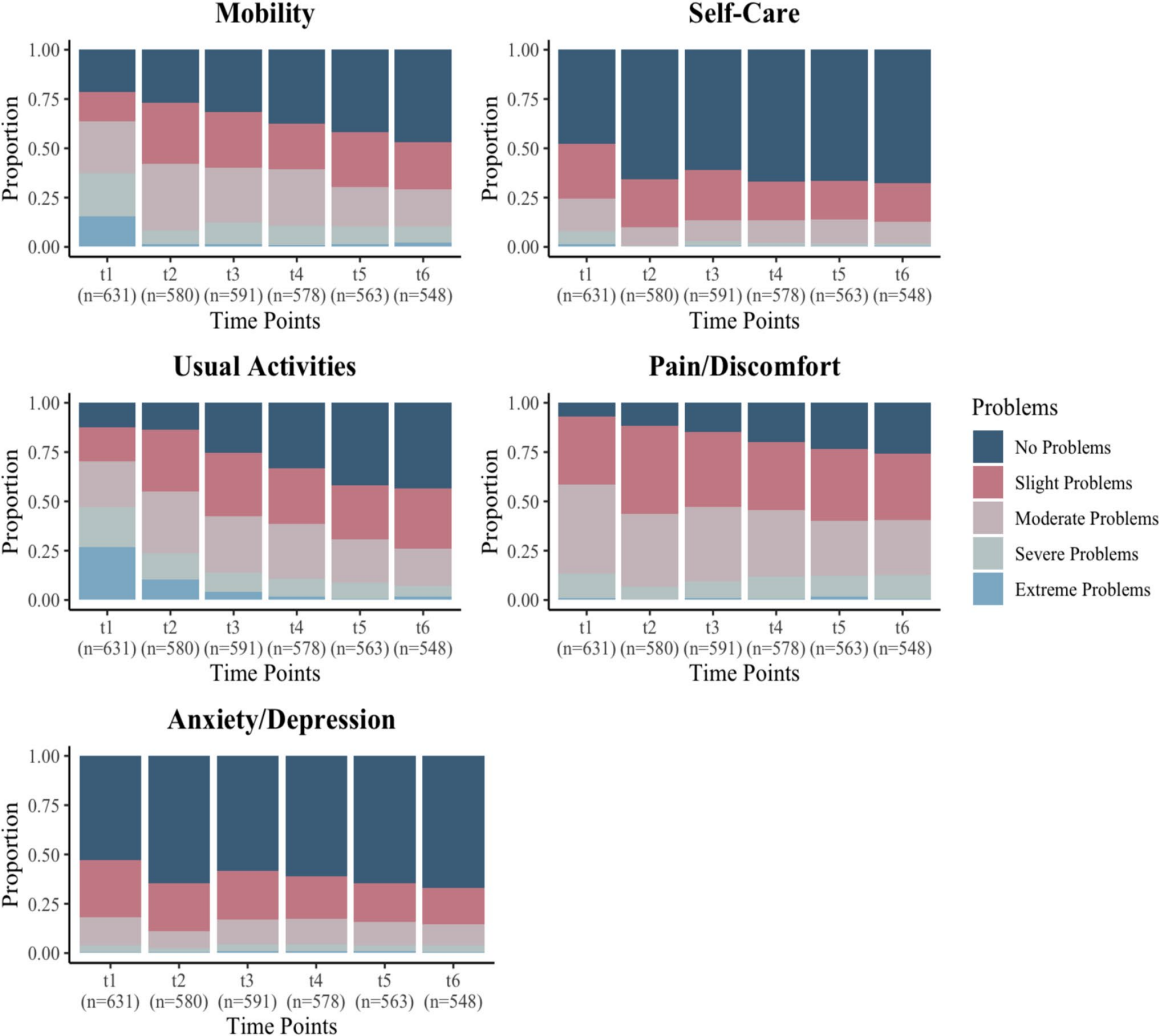
Change in the five dimensions of the EQ-5D-5L from admission (t1) to 78 weeks after discharge from inpatient rehabilitation (t6)

Figure 1 shows the proportions and changes in the five dimensions of the EQ-5D-5L, with the respective numbers of available data at different time points. At baseline (t1), patients reported the greatest difficulties with *Mobility* and *Usual Activities*. These dimensions demonstrated the highest degree of improvements, with a notable reduction in *extreme problems* between admission and discharge (t2). *Pain/Discomfort* also showed significant improvements over time. However, it became evident that, in comparison to the other dimensions, this dimension still had the smallest proportion of patients reporting "no problems" even 78 weeks after discharge from inpatient rehabilitation. Positive changes were observed in all five dimensions from admission to 78 weeks after discharge (t6), particularly in *Self-Care* and *Anxiety/Depression,* where more than 60% of patients reported having *no problems* at discharge. This proportion remained stable at further time points, showing only a slight decrease at 12 weeks after discharge (t3) before nearly returning to discharge levels (t2) by 78 weeks after discharge (t6).

**Fig. 2:**
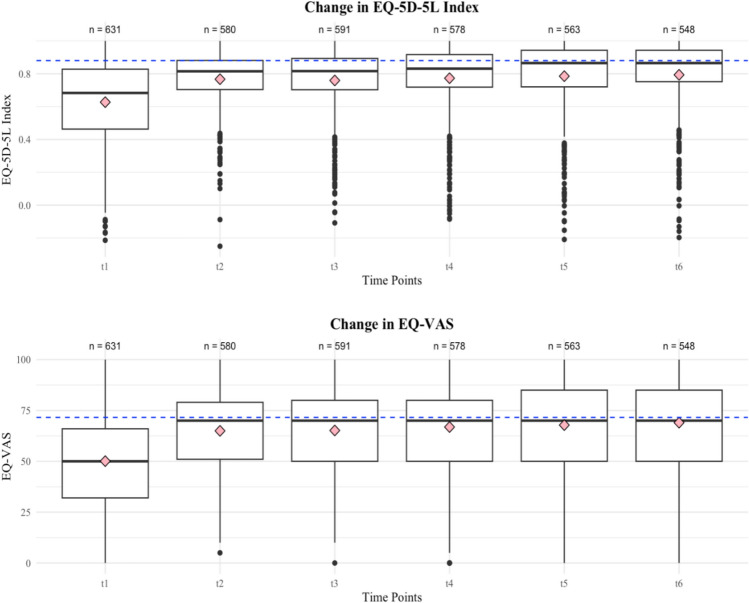
Change in EQ-5D Index and EQ-VAS. Blue line: Norm value of the German general population (EQ-5D Index: 0.88; EQ-VAS: 71.6) [19]

Figure 2 illustrates the change in the EQ-VAS and EQ-5D Index from admission to inpatient rehabilitation to 78 weeks after discharge. Both variables showed a positive trend from admission (t1) to discharge (t2), continuing to increase until 78 weeks after discharge (t6). Supplementary Table 2 shows the boxplot values, where the mean EQ-VAS increased by 38%, and the EQ-5D Index by 25.4%. The median EQ-VAS remained stable from t2 to t6, while the median EQ-5D Index showed a slight upward trend, stabilizing around 0.8. The Interquartile Range (IQR) remained relatively constant after discharge, indicating consistent variability in responses. The persistent presence of outliers in the EQ-5D Index suggests that some patients reported significantly lower quality of life.

### Growth Model: Change Over Time in Current Overall Health Status (EQ-VAS)

Table 2 shows the results of the growth model. In the unconditional Model 1, the ICC of the intercept variance was approximately 40% for patients and 0.7% for clinics, indicating the variance in EQ-VAS between patients and between clinics. The average EQ-VAS over all time points and for all patients was 63.26 (*p* < 0.05). In Model 2, the level-1 predictor time was added as a fixed effect to assess the estimate of the impact of time on the outcome. The results indicated a gradual and consistent increase in EQ-VAS over time. For instance, at discharge (t2), the EQ-VAS increased by an average of 14.9 units compared to admission (t1), with all time points showing statistically significant increases (*p* < 0.05). With Model 3, the aim was to add the EQ-VAS at baseline (t1) as a fixed effect to estimate the impact on the outcome over time. The result showed that the baseline EQ-VAS had a significant influence on EQ-VAS over time for 0.46 points (*p* < 0.05).

**Table 2:** Three-Level Growth Modeling

Effect	Model 1 (unconditional model)		Model 2 (basic time model)		Model 3	
	Estimate	95%-CI	Estimate	95%-CI	Estimate	95%-CI
Fixed Effects						
Intercept	63.26*	[61.49;65.01]	49.78*	[47.79; 51.74]	27.07*	[24.43; 29.76]
t2			14.90*	[13.23; 16.51]	14.86*	[13.22; 16.49]
t3			15.07*	[13.44; 16.70]	15.07*	[13.44; 16.70]
t4			16.60*	[14.95; 18.23]	16.53*	[14.89; 18.17]
t5			17.82*	[16.16; 19.47]	17.83*	[16.17; 19.48]
t6			18.76*	[17.09; 20.43]	18.74*	[17.08; 20.41]
EQ-VAS at t1					0.46*	[0.41; 0.50]

Cov parameter	Variance	SD	Variance	SD	Variance	SD
Random effects						
Error variance						
Intercept (ID)	178.1	13.35	186	13.64	98.2	9.9
Intercept (Clinic)	2.98	1.7	2.18	1.5	0.38	0.61
Residual	260.92	16.15	209.72	14.5	209.6	14.5
Model Fit						
AIC	30,325.30		29,706.20		29,390.50	

t2: discharge from inpatient rehabilitation; Follow-up after discharge from inpatient rehabilitation: t3: 12 weeks, t4: 26 weeks, t5: 52 weeks, t6: 78 weeks

Cov Parameter: Covariance parameter; SD: Standard deviation

AIC: Akaike information criterion

^*^Significant *p* values (*p* value < 0.05); 95%-CI 95% confidence interval

As shown in Supplementary Table 3, Model 4a analyzed the known influential factors from the literature, including contextual, biopsychosocial, and environmental factors. In Model 4a, baseline EQ-VAS had a slightly positive but significant effect on the outcome (0.34, *p* < 0.1; 95%-CI 0.29–0.39). Interestingly, male patients showed lower EQ-VAS scores compared to their female counterparts (−2.16, *p* < 0.1; 95%-CI −4.18 to −0.14). Age also played a role, with older individuals experiencing a decline in their current overall health status (−0.14, *p* < 0.1; 95%-CI −0.22 to −0.07). The impact of physical and emotional well-being was evident, as those experiencing more pain under stress (−0.07, *p* < 0.05; 95%-CI −0.12 to −0.03), more pain at rest (−0.07, *p* < 0.05; 95%-CI −0.12 to −0.01), poorer sleep quality (−0.05, *p* < 0.05; 95%-CI −0.08 to −0.01), or those with a higher likelihood of anxiety based on the PHQ-4 had significantly worse EQ-VAS scores (−3.28, *p* < 0.05; 95%-CI −5.98 to −0.58).

Furthermore, the presence of two comorbidities (−2.37, *p* < 0.1; 95%-CI −5.03 to 0.30) was associated with a negative impact on perceived health. Individuals who reported being partially burdened (−2.41, *p* < 0.05; 95%-CI −4.63 to −0.19) or very burdened (−2.55, *p* < 0.1; 95%-CI −5.11 to 0.01) by visible consequences of the accident (e.g., scars) had significantly lower EQ-VAS scores. Patients with higher self-efficacy (−1.30, *p* < 0.1; 95%-CI −2.7 to 0.1) and better life satisfaction pre-accident (−0.04, *p* < 0.05; 95%-CI −0.08 to 0) also experienced a negative impact on EQ-VAS. On a more positive note, having a higher income, specifically 3200 euros or more per month, was linked to better EQ-VAS scores (3.89, *p* < 0.05; 95%-CI 1.21–6.57).

In Supplementary Table 4, the results of Model 4b are presented. Compared to Model 4a, Model 4b has still similar effects regarding the variables mentioned above. Notably, patients who expressed a strong belief in RTW showed significantly better EQ-VAS scores (4.01, *p* < 0.05; 95%-CI 0.68–7.35). Additionally, individuals who had experienced a stressful life event in the past 12 months reported significantly higher EQ-VAS scores (2.88, *p* < 0.05; 95%-CI 0.89–4.87), as did those living with others (2.15, *p* < 0.1; 95%-CI −0.31 to 4.6).

A comprehensive overview of all the variables used is provided in Supplementary Table 5.

Table 3 presents the average EQ-VAS according to diagnoses at admission (t1), discharge (t2), and 78 weeks after discharge (t6). The greatest improvement in EQ-VAS was observed between admission and discharge from the inpatient rehabilitation across all injury types, except for multiple injuries with severe manifestations (Injury type 8), where the EQ-VAS slightly decreased after discharge. For other diagnoses, EQ-VAS increased by less than 7% after discharge, except for severe chest or abdominal injuries with organ involvement (Injury type 3) and injuries to the great vessels (Injury type 2), which showed improvements of 65.5% and 57%, respectively, from t1 to t6.

**Table 3:** Average EQ-VAS of different injury types

Time	Injury type 1	Injury type 2	Injury type 3	Injury type 4	Injury type 5	Injury type 6	Injury type 7	Injury type 8
t1	53.0	43.0	42.7	50.3	48.8	53.5	48.2	42.8
t2	65.7	52.5	57.5	65.5	64.1	69.5	65.8	61.5
t6	66.8	67.5	70.7	69.4	68.3	71.9	68.5	60.3

t1: admission, t2: discharge from inpatient rehabilitation, t6: 78 weeks after discharge

Injury type 1: Extensive or deep injuries of the skin and soft tissue mantle; amputation injuries; muscle compression syndromes (compartment syndromes); thermal or chemical damage; Injury type 2: Injuries to the great vessels; Injury type 3: Severe chest or abdominal injuries with organ involvement including kidneys or urinary tract; Injury type 4: Complex fractures of the large tubular bones, especially multiple or open fractures; Injury type 5: Severe injuries to large joints; Injury type 6: Severe injuries to the hand; Injury type 7: Complex fractures of the facial skull and torso skeleton; Injury type 8: Multiple injuries with severe manifestations [16]

## Discussion

This study captured comprehensive data on functioning, disability, contextual factors, and injury specifics across ten German clinics, exploring the influence of these factors on the current overall health status. The primary objective of this study was to assess the change over time in the current overall health status among patients with traumatic musculoskeletal injuries. Throughout the study period, a consistent and significant increase in EQ-VAS was observed, indicating overall improvement in patient outcomes. Notably, baseline EQ-VAS had a substantial influence on subsequent changes, suggesting that initial health pre-rehabilitation status plays a critical role in recovery trajectories. Additionally, several factors were found to significantly impact the change over time in EQ-VAS, with different types of injuries exhibiting varying levels of improvement. This highlights the complex interplay of individual and injury-specific variables in determining rehabilitation outcomes.

One notable aspect is the predominance of male patients in the cohort, which can be attributed to the nature of work-related injuries. These injuries commonly occur in sectors such as production, manufacturing, construction, logistics, and security, where men are overrepresented. In Germany, the proportion of women in these sectors was less than one-third in 2023 [34].

In our population, the average interval between discharge from acute care and admission to inpatient rehabilitation was 37 days. Although no statistically significant association with our outcomes was observed, it is important to highlight that prolonged inactivity, bed rest, and immobility may influence recovery outcomes. These factors can have detrimental effects on the musculoskeletal system, underscoring the importance of timely initiation of rehabilitation interventions. Rehabilitation should ideally start as soon as possible following acute care to restore patients' ability to perform activities of daily living, tailored to their individual medical needs [35]. Although most patients received treatment after discharge from the acute clinic, 17% did not, highlighting a gap in care. Early initiation of inpatient rehabilitation could reduce healthcare costs by minimizing nursing care requirements, shortening hospital stays, and lowering disability rates [35]. A German study about a rehabilitation program for work-related injuries of the hand found that an early start of inpatient rehabilitation leads to better functional outcomes for the patient compared to a later start [36]. In contrast to the national average of 21.6 days for musculoskeletal rehabilitation in Germany, our cohort had an average stay of 44.4 days, likely reflecting more severe cases [37].

The descriptive analysis revealed that upon admission to inpatient rehabilitation, nearly all patients reported difficulties with *Usual Activities* and *Pain/Discomfort,* consistent with findings in another study from Spain [38]. This finding is unsurprising, given that musculoskeletal injuries typically result in persistent pain and impaired daily function [5]. While rehabilitation positively impacted all dimensions by discharge, the improvements in *Usual Activities* and *Pain/Discomfort* remained moderate, with more than half of the patients still experiencing issues. This suggests that these dimensions require focused attention during and after rehabilitation. A study from Norway found out that more than two years post-trauma, the majority still had pain, suggesting that effective pain management is crucial for improving long-term recovery and a better EQ-VAS after trauma [39]. Furthermore, the dimensions *Self-Care* and *Anxiety/Depression* exhibited a temporary decline 12 weeks after discharge. This could be due to the transition from the structured, supportive environment of the clinic to the challenges of daily life at home, where accessibility and support may be limited. However, both dimensions improved over time, indicating that this dip is likely temporary.

The EQ-5D Index and the EQ-VAS both showed positive trends, but the EQ-5D Index provided a more detailed reflection of HRQoL due to its multiple dimensions. The narrowing of the EQ-5D Index plots over time suggests a convergence in patient outcomes, despite the presence of outliers. Future research could benefit from a longitudinal analysis of the EQ-5D Index to gain deeper insights into patient recovery. Although the EQ-VAS values in our study population increased over time, they remained below the normative values of the German general population. The literature presents varying average EQ-VAS scores for Germany, with one study reporting an average of 71.6 [19] and another reporting 84.3 [15]. However, despite these differences, both values are consistently higher than the average EQ-VAS scores observed in our study population, even at the end of the follow-up period, i.e., 78 weeks after discharge from first inpatient rehabilitation. These findings align with those of a Spanish cohort study, which indicates that while the HRQoL of trauma patients improves in the initial years following the trauma, it never returns to the reference levels observed for the general population [38].

The ICC analysis indicated that less than one percent of the variance was attributable to the clinic, suggesting that patient-specific factors are more influential. Model 2 confirmed the positive impact of inpatient rehabilitation and the beneficial role of time in enhancing recovery and well-being. The significant influence of the baseline EQ-VAS underscores the importance of initial assessments in predicting rehabilitation outcomes. Early identification of patients at risk could enable more tailored interventions, potentially improving long-term HRQoL.

Regarding post-acute care treatments, the findings are ambivalent concerning their impact on outcomes, with the majority of results showing no statistical significance. Nonetheless, it is fundamentally important to recognize that outpatient treatment following discharge from acute care can influence long-term HRQoL, and any form of treatment is preferable to none at all. Early identification of impairments and the creation of informed care transitions—such as from acute care to outpatient services and subsequently back to inpatient rehabilitation—are essential to optimize the rehabilitation process and improve HRQoL. Future research should focus on these areas to further enhance patient outcomes.

Living with family generally has a positive effect on HRQoL for injured patients, a finding consistent with our results [30]. However, our study also revealed that the feeling of support from family negatively impacted EQ-VAS, possibly due to the strain caregiving imposes on family dynamics. This is supported by existing literature, which documents the physical, psychological, and social challenges families face when caring for an injured member. These challenges can aggravate health issues, increase financial burdens, and lead to emotional instability. To mitigate these effects, interventions that alleviate pain and discomfort are essential, along with reinforcing community support programs, including counseling and support groups [30]. Interestingly, support from colleagues and the workplace had a positive effect on EQ-VAS, highlighting not only the importance of social support but also the critical role that social participation and reintegration play in the recovery process.

The supplementary models indicated that comorbidities and severe injuries negatively affected EQ-VAS, supported by other results in literature [40, 41]. Anxiety and depression also emerged as significant factors, consistent with other studies that link these conditions to trauma and long-term quality of life challenges [40, 42, 43]. Other studies which focused on psychiatric comorbidity and anxiety for trauma patients found that anxiety and depression do not independently predict poor HRQoL, but are mediated by baseline health status and severity of injury [31]. Another paper found out that depression and anxiety independently have a negative impact on the HRQoL [44]. Our results show that the baseline EQ-VAS and severe injuries have a negative effect on the outcome, hence further research can be conducted to analyze the relation between these different variables in our specific study population. To significantly enhance EQ-VAS outcomes, the implementation of a care team that includes psychiatrists, psychologists, therapists, and traumatologists within inpatient rehabilitation is crucial. While interdisciplinary teams are already a key component of rehabilitation care, this approach emphasizes the importance of specialized collaboration to proactively identify and address patients at risk for anxiety and depression. By screening these patients early, the team can deliver more precise interventions, thereby further improving EQ-VAS outcomes [31].

Ongoing legal disputes related to the accident were reported by a quarter of our study population before their admission to inpatient rehabilitation. Such disputes can distract patients and hinder their recovery, reducing their motivation to recover quickly, often in pursuit of better outcomes in their legal cases, such as compensation for personal suffering [29]. The same applies to potential pension claims. On the other hand, patients who feel responsible for the accident may experience guilt and shame, further lowering their current overall health status [45]. Providing patients with a realistic outlook on the likelihood of success in their legal processes, alongside refocusing them on recovery with the support of rehabilitation managers and physicians, may be beneficial. Psychological assistance is also crucial to help patients process feelings of guilt.

Our results show that self-efficacy—defined as an individual's belief in their ability to achieve and engage in their goals [46]—had a negative effect on EQ-VAS in our study population. This is contrary to other literature, which typically associates self-efficacy with positive outcomes [46]. One possible explanation for this discrepancy is that patients with high self-efficacy may overestimate their ability to cope with their injury, leading to frustration when their recovery does not meet expectations. Additionally, these individuals may be less likely to seek help, putting additional pressure on themselves and resulting in lower EQ-VAS scores.

The study also highlighted the importance of non-medical factors on recovery outcomes. For instance, visible consequences of the accident, such as scars, were burdening for a third of our study population and had a significant negative impact on EQ-VAS. This underscores the need for psychological support to help patients manage distress associated with visible consequences. For example, offering timely information about available interventions, such as plastic surgery, could enhance patients' sense of hope and optimism about their overall recovery.

Lastly, our findings show a significant positive effect of social burden on EQ-VAS. Although this may seem counterintuitive, it is possible that patients with social responsibilities feel a strong motivation to recover quickly and effectively, driven by the awareness that others rely on them. This sense of responsibility might provide a powerful motivator and give patients a sense of purpose.

A key strength of this study is the extended follow-up period of 78 weeks, which exceeds the typical 12-month mark of most studies in this area. This longer follow-up provides valuable insights into the long-term outcomes of patients with traumatic musculoskeletal injuries after work-related accidents. Additionally, the study’s broad focus on various influential factors beyond medical ones highlights the importance of considering functioning, disability, and contextual and environmental factors.

However, a limitation of this study is that the potentially influencing variables were only assessed at baseline and not subsequently monitored during the follow-up period. Furthermore, not all participants completed every follow-up assessment, which introduces the possibility of attrition bias. However, we addressed this using multilevel growth modeling, which is specifically designed to handle missing data and unequally spaced time points. This method allows for the inclusion of participants with partially missing data and is considered a robust statistical approach in longitudinal research. In addition, treatment during inpatient rehabilitation was not standardized across clinics. The first inpatient trauma rehabilitation in Germany is not a uniform intervention but is individualized according to injury severity, recovery trajectory, and medical decision-making. While this variability may be perceived as a limitation, it reflects real-world practice and thus enhances the external validity of our findings. Moreover, individuals with language barriers were unable to participate, as the comprehensive assessment conducted at the time of rehabilitation admission required a strong proficiency in German. The discharge from the initial inpatient rehabilitation following the accident constituted the baseline for the subsequent 78-week follow-up period. This indicates that the interval between the accident and the patient's discharge from the initial inpatient rehabilitation varied among individuals. If the day of the accident had been taken as the starting point for calculating the 78-week follow-up period, the results may have differed. Nevertheless, establishing a direct connection to the accident date is difficult due to the inherent variability in the duration of acute care, the onset of treatment, and the length of rehabilitation. The date of discharge from the inpatient rehabilitation was selected with the intention of establishing a baseline status quo, given that it can be reasonably assumed that patients are in a relatively similar condition at this point in their recovery. Lastly, it should be noted that, due to the absence of a randomized control group, the observational design of this study does not allow for causal inferences regarding the effect of inpatient rehabilitation on changes in health-related quality of life.

Finally, in light of these results, several implications for clinical practice and rehabilitation planning emerge, particularly with regard to individualized, biopsychosocial interventions. These findings underscore the need for tailored, biopsychosocial rehabilitation strategies. Based on the identified predictors, early integration of pain management, psychological support (e.g., for anxiety, sleep disturbances, and visible consequences as scars), and structured peer involvement may be beneficial. In addition, assistive device provision and family engagement should be considered to support long-term recovery and reintegration. Such individualized approaches may also inform future rehabilitation policy.

## Conclusion

This study provides valuable insights into the change over time in EQ-VAS in patients with traumatic musculoskeletal work-related injuries undergoing inpatient rehabilitation. The findings indicate positive developments in current overall health status during the rehabilitation period. Factors such as injury type, comorbidities, and treatment types significantly influenced recovery trajectories, underscoring the importance of personalized interventions. Notably, the study emphasizes the relevance of the ICF framework in capturing the multidimensional aspects of patient recovery. The study also reveals the complexities surrounding social and psychological factors, such as family support, self-efficacy, and visible consequences, which can either facilitate or hinder recovery. These findings suggest the need for comprehensive, multidisciplinary approaches that address both medical and biopsychosocial aspects of rehabilitation to optimize patient outcomes. The findings of this study demonstrate that significant positive changes occur between admission in inpatient rehabilitation and discharge in terms of the current overall health status. However, this progress tends to stagnate over time, and even more than 1.5 years after the traumatic event, the average current overall health status does not reach the levels observed in the general population. This highlights the crucial role that rehabilitation plays in the recovery process. Additionally, it emphasizes the need for early identification of at-risk patients through a holistic approach, allowing for targeted interventions on time that could enhance long-term recovery outcomes.

In summary, measurable improvements were observed in both current overall health status and HRQoL throughout the study period. Future research should continue to examine recovery after work-related injuries using longitudinal designs and integrative models. In particular, studies that systematically incorporate the biopsychosocial perspective—aligned with the ICF framework—will be essential to further advance patient-centered rehabilitation strategies.

## Supplementary Information

Below is the link to the electronic supplementary material.

Supplementary file1 (DOCX 340 KB)

## Data Availability

The datasets generated during and/or analyzed during the current study are available from the corresponding author on reasonable request.

## Abbreviations

AIC: Akaike information criterion
EQ-5D-5L: EuroQol -5 dimensions -5 levels
HRQoL: Health-related quality of life
ICC: Intraclass correlation coefficient
ICF: International Classification of Functioning, Disability, and Health
ILO: International Labour Organization
IQR: Interquartile range
PHQ-4: Patient Health Questionnaire
REDCap: Research electronic data capture
RTW: Return to work
SGB: Social Code Book
VAS: Visual analogue scale
WHO: World Health Organization
WHODAS: WHO Disability Assessment Schedule
WS: Working sector
WHO: World Health Organization
WHODAS: WHO Disability Assessment Schedule
