# Supplementary material for: Changes and Determinants of Health-Related Quality of Life in Patients with Work-Related Traumatic Musculoskeletal Injuries: A Longitudinal Analysis of HRQoL and the EQ-VAS
Source: J Occup Rehabil. 2025 Jun 23;36(3):699–712. doi: 10.1007/s10926-025-10304-4 (PMC13364787; doi:10.1007/s10926-025-10304-4)
Supplement: Supplementary file 1 — Supplementary file1 (DOCX 340 KB) [file 10926_2025_10304_MOESM1_ESM.docx]

# Supplementary Information

## Supplementary Tables

**Supplementary Table 1** Sociodemographic and injury-specific data at admission to first inpatient rehabilitation after trauma (t1) (n = 698).: m = mean, SD = standard deviation

|  | N (%) |
| --- | --- |
| **Exposure to special environmental conditions (e.g., noise, drafts, cold, moisture) at workplace *** | 426 (61%) |
| **Clinics** |  |
| Clinic 1 | 201 (29%) |
| Clinic 2 | 95 (14%) |
| Clinic 3 | 22 (3%) |
| Clinic 4 | 59 (8%) |
| Clinic 5 | 88 (13%) |
| Clinic 6 | 111 (16%) |
| Clinic 7 | 54 (8%) |
| Clinic 8 | 12 (2%) |
| Clinic 9 | 39 (6%) |
| Clinic 10 | 17 (2%) |
| **Injury localization** |  |
| One | 502 (72%) |
| Two | 134 (19%) |
| Three or more | 62 (9%) |
| **Complication in healing process *** | 28 (4%) |
| **Comorbidities** |  |
| None | 207 (30%) |
| One | 204 (29%) |
| Two | 127 (18%) |
| Three or more | 160 (23%) |
| **Smoking status *** | 216 (31%) |
| **Heavy drinking** |  |
| No risk | 425 (61%) |
| Increased risk | 100 (14%) |
| Risk | 119 (17%) |
| No information | 54 (8%) |
| **Visible consequences of the accident are burdening (e.g., scars)** |  |
| Does not apply | 447 (64%) |
| Partially applies | 142 (20%) |
| Applies | 109 (16%) |
| **Pain at rest, m(SD); scale: 0-100** | 24.2 (21.0) |
| **Pain under stress, m(SD); scale: 0-100** | 47.0 (22.7) |
| **Sleep, m(SD); scale: 0-100** | 48.8(27.4) |
| **Signs of anxiety (PHQ-4) *** | 101 (15%) |
| **Signs of depression (PHQ-4) *** | 161 (23%) |
| **Regular engagement in sports before the accident** |  |
| No sport | 327 (47%) |
| Below the recommended level | 105 (15%) |
| As recommended | 266 (38%) |
| **Subjective prognosis on return to work** |  |
| Unlikely | 59 (8%) |
| Not sure | 243 (35%) |
| Very likely | 396 (57%) |
| **Ongoing legal disputes related to accident *** | 179 (26%) |
| **Demand for pension claim *** | 14 (2%) |
| **Duration of inpatient rehabilitation m, (SD)** | 44.4 (38.1) |
| **Rehabilitation plan *** | 406 (58%) |
| **Feeling of support by family and friends** |  |
| Not supported to moderately supported | 84 (12%) |
| Supported | 198 (28%) |
| Strongly supported | 416 (60%) |
| **Feeling of support by company/colleagues** | |
| Not supported to moderately supported | 301 (43%) |
| Supported | 206 (30%) |
| Strongly supported | 191 (27%) |
| **Accessible housing *** | 269 (39%) |
| *Dichotomously coded variable (yes / no), only the result (n and %) is shown in relation to the “yes” response option  **Pain at rest / pain under stress:** 0 = no pain to 100 = excruciating pain  **Sleep:** 0 = very good to 100 = very bad | |

**Supplementary Table 2** Boxplot Values

| Boxplot Values for the EQ-5D Index | | | | | | |
| --- | --- | --- | --- | --- | --- | --- |
| **Time** | **Minimum** | **Q1** | **Median** | **Q3** | **Maximum** | **Mean** |
| t1 | - 0.21 | 0.46 | 0.68 | 0.83 | 1.00 | 0.63 |
| t2 | - 0.25 | 0.70 | 0.82 | 0.88 | 1.00 | 0.77 |
| t3 | - 0.11 | 0.70 | 0.82 | 0.89 | 1.00 | 0.76 |
| t4 | - 0.09 | 0.72 | 0.83 | 0.92 | 1.00 | 0.77 |
| t5 | - 0.21 | 0.72 | 0.87 | 0.94 | 1.00 | 0.79 |
| t6 | - 0.20 | 0.75 | 0.87 | 0.94 | 1.00 | 0.79 |
| Boxplot Values for the EQ-5D VAS | | | | | | |
| **Time** | **Minimum** | **Q1** | **Median** | **Q3** | **Maximum** | **Mean** |
| t1 | 0 | 32 | 50 | 66 | 100 | 50.09 |
| t2 | 5 | 51 | 70 | 79 | 100 | 64.97 |
| t3 | 0 | 50 | 70 | 80 | 100 | 65.16 |
| t4 | 0 | 50 | 70 | 80 | 100 | 66.86 |
| t5 | 0 | 50 | 70 | 85 | 100 | 67.81 |
| t6 | 0 | 50 | 70 | 85 | 100 | 69.00 |
| t1: admission; t2: discharge from inpatient rehabilitation  Follow-up after discharge from inpatient rehabilitation: t3: 12 weeks, t4: 26 weeks, t5: 52 weeks, t6: 78 weeks  Q1: 25^th^ percentile, Q3: 75^th^ percentile from the interquartile range (IQR) | | | | | | |

**Supplementary Table 3** Three-Level Growth Model 4a with possible influencing factors

| **Term** | | **Estimate** | **P-value** | **95% - CI** |
| --- | --- | --- | --- | --- |
| **Intercept** |  | 60.13 | **0.00** | [48.87; 71.38] |
| **t2** |  | 14.87 | **0.00** | [13.23; 16.51] |
| **t3** |  | 15.05 | **0.00** | [13.42; 16.68] |
| **t4** |  | 16.50 | **0.00** | [14.86; 18.14] |
| **t5** |  | 17.74 | **0.00** | [16.09; 19.39] |
| **t6** |  | 18.70 | **0.00** | [17.03; 20.36] |
| **Age at admission** |  | -0.14 | **0,00** | [-0.22; -0.07] |
| **Gender** *[Ref = Female]* | Male | -2.16 | **0,04** | [-4.18; -0.14] |
| **BMI** |  | -0.04 | 0,68 | [-0.20; 0.13] |
| **Comorbidities** *[Ref = None]* | One | -1.45 | 0,20 | [-3.66; 0.76] |
|  | Two | -2.37 | **0,08** | [-5.03; 0.30] |
|  | Three or more | -1.77 | 0,18 | [-4.37; 0.83] |
| **Income** *[Ref = Under 1,700* *€]* | Between 1,700 € and 2,300 € | 1.08 | 0,43 | [-1.58; 3.74] |
|  | Between 2,300 € and 3,200 € | 0.92 | 0,51 | [-1.81; 3.64] |
|  | 3,200 € and more | 3.89 | **0,00** | [1.21; 6.57] |
|  | No information | 1.17 | 0,47 | [-2.02; 4.36] |
| **Type of injury** *[Ref = Not this type]* | Injury type 1 | -0.17 | 0,94 | [-4.32; 3.98] |
|  | Injury type 2 | -1.48 | 0,78 | [-11.88; 8.91] |
|  | Injury type 3 | -0.53 | 0,80 | [-4.63; 3.56] |
|  | Injury type 4 | -1.35 | 0,35 | [-4.17; 1.48] |
|  | Injury type 5 | -0.16 | 0,91 | [-2.97; 2.64] |
|  | Injury type 6 | 0.12 | 0,96 | [-4.16; 4.4] |
|  | Injury type 7 | 0.17 | 0,91 | [-2.71; 3.05] |
|  | Injury type 8 | -3.84 | **0,07** | [-7.94; 0.26] |
| **Complication in healing process** *[Ref = Not present]* | Complication present | 1.21 | 0.62 | [-3.57; 5.99] |
| **Severity of injury** *[Ref = 1V]* | >2S | -0.44 | 0.86 | [-5.31; 4.43] |
|  | >2V | -0.22 | 0.94 | [-5.80; 5.36] |
|  | 1S | -1.93 | 0.10 | [-4.22; 0.35] |
|  | 2S | -2.66 | 0.14 | [-6.20; 0.88] |
|  | 2V | 1.73 | 0.31 | [-1.58; 5.04] |
| **Treatment type pre-admission** *[Ref = Not this treatment]* | Physiotherapy | 0.93 | 0.55 | [-2.15; 4.02] |
|  | Occupational therapy | -0.98 | 0.41 | [-3.33; 1.37] |
|  | Massage/lymphatic drainage | -0.43 | 0.66 | [-2.34; 1.48] |
|  | Medical training therapy | -3.39 | **0.02** | [-6.26; -0.53] |
|  | Psychological therapy | 1.32 | 0.49 | [-2.40; 5.04] |
|  | Other | 2.10 | 0.21 | [-1.19; 5.39] |
|  | None | 1.71 | 0.37 | [-2.03; 5.44] |
| **Pain under stress** |  | -0.07 | **0.00** | [-0.12; -0.03] |
| **Pain at rest** |  | -0.07 | **0.01** | [-0.12; -0.01] |
| **Smoking status** *[Ref = Not smoking]* | Smoking | -1.61 | 0.12 | [-3.64; 0.41] |
| **Heavy drinking** *[Ref = No risk]* | Increased risk | 1.80 | 0.15 | [-0.65; 4.25] |
|  | Risk | 0.84 | 0.49 | [-1.53; 3.2] |
|  | No information | -3.08 | **0.08** | [-6.51; 0.36] |
| **Visible consequences of the accident are burdening (e.g., scars)**  *[Ref = Does not apply]* | Partially applies | -2.41 | **0.03** | [-4.63; -0.19] |
|  | Applies | -2.55 | **0.05** | [-5.11; 0.01] |
| **Sleep** |  | -0.05 | **0.01** | [-0.08; -0.01] |
| **General: Looking after health** *[Ref = Slightly]* | Moderately | 1.54 | 0.33 | [-1.53; 4.6] |
|  | Strongly | 1.83 | 0.27 | [-1.41; 5.06] |
| **Feeling of support by family and friends** *[Ref = Not supported to moderately supported]* | Supported | -1.15 | 0.47 | [-4.28; 1.97] |
|  | Strongly supported | -1.04 | 0.53 | [-4.27; 2.19] |
| **Feeling of support by company/colleagues** *[Ref = Not supported to moderately supported]* | Supported | 1.93 | **0.08** | [-0.21; 4.07] |
|  | Strongly supported | 2.15 | **0.07** | [-0.21; 4.51] |
| **Acceptance of the new situation by family** *[Ref = Not accepted to moderately accepted]* | Having no family | 0.31 | 0.90 | [-4.47; 5.08] |
|  | Accepted | 1.61 | 0.41 | [-2.21; 5.43] |
|  | Strongly accepted | 2.07 | 0.27 | [-1.62; 5.76] |
|  | No information | 2.70 | 0.29 | [-2.29; 7.68] |
| **School education** *[Ref = Graduation up to grade 12]* | No graduation | 2.80 | 0.29 | [-2.39; 8.00] |
|  | Graduation up to grade 9 | -1.71 | 0.15 | [-4.02; 0.6] |
|  | Graduation up to grade 10 | -1.01 | 0.38 | [-3.23; 1.22] |
| **Self-efficacy** |  | -1.30 | **0.07** | [-2.7; 0.1] |
| **Signs of depression: PHQ-4** *[Ref = No]* | Yes | -0.24 | 0.84 | [-2.5; 2.03] |
| **Signs of anxiety: PHQ-4** *[Ref = No]* | Yes | -3.28 | **0.02** | [-5.98; -0.58] |
| **Life satisfaction pre-accident** |  | -0.04 | **0.04** | [-0.08; 0] |
| **EQ-VAS at baseline (t1)** |  | 0.34 | **0.00** | [0.29; 0.39] |
| **WHODAS-Score** |  | -0.04 | 0.12 | [-0.09; 0.01] |
| **AIC** 29,338.4 | | | | |
| **Interpretation example:** **1.** Time: The estimate of 18.70 for *t6* indicates that, on average, patients reported EQ-VAS scores 18.70 points higher 78 weeks after discharge compared to baseline (t1), assuming all other variables remain constant. **2**. Income: An estimate of 3.89 for the category *3,200 € and more* indicates that, on average, patients in this income group reported EQ-VAS scores that were 3.89 points higher over time than those with a monthly household income under 1,700 €, controlling for all other variables in the model.  **Bold values indicate p-values < 0.1**; 95% - CI: 95 % confidence interval  AIC: Akaike information criterion  Explanations: t2: discharge from inpatient rehabilitation, t3: 12 weeks, t4: 26 weeks, t5: 52 weeks, t6: 78 weeks after discharge  **Injury types:** Injury Type 1: Extensive or deep injuries of the skin and soft tissue mantle; amputation injuries; muscle compression syndromes (compartment syndromes); thermal or chemical damage; Injury Type 2: Injuries to the great vessels; Injury Type 3: Severe chest or abdominal injuries with organ involvement including kidneys or urinary tract; Injury Type 4: Complex fractures of the large tubular bones, especially multiple or open fractures; Injury Type 5:  Severe injuries to large joints; Injury Type 6: Severe injuries to the hand; Injury Type 7: Complex fractures of the facial skull and torso skeleton; Injury Type 8: Multiple injuries with severe manifestations  **Severity of injury:** 1V = one diagnosis: no severe injury, 1S = one diagnosis: severe (S) injury, 2V = two diagnoses: no severe injury, 2S = two diagnoses: at least one severe injury, >2V = more than 2 diagnoses, no severe injury, >2S = more than 2 diagnoses, at least one severe injury  **Pain at rest / Pain under stress:** 0 = no pain to 100 = excruciating pain  **Sleep**: 0 = very good to 100 = very bad  **PHQ-4:** Patient Health Questionnaire  **WHODAS:** WHO Disability Assessment Schedule, 0 = no disability to 100 = complete disability | | | | |

**Supplementary Table 4** Three-Level Growth Model 4b with potential influencing factors

| **Term** | | **Estimate** | **P-value** | **95% - CI** |
| --- | --- | --- | --- | --- |
| **Intercept** |  | 62.96 | **0.00** | [37.07; 88.84] |
| **t2** |  | 14.87 | **0.00** | [13.23; 16.51] |
| **t3** |  | 15.06 | **0.00** | [13.43; 16.68] |
| **t4** |  | 16.49 | **0.00** | [14.85; 18.13] |
| **t5** |  | 17.74 | **0.00** | [16.09; 19.4] |
| **t6** |  | 18.68 | **0.00** | [17.02; 20.35] |
| **Age at admission** |  | -0.15 | **0.00** | [-0.23; -0.07] |
| **Gender** *[Ref = Female]* | Male | -0.45 | 0.71 | [-2.83; 1.92] |
| **BMI** |  | -0.03 | 0.73 | [-0.2; 0.14] |
| **Comorbidities** *[Ref = None]* | One | -1.51 | 0.18 | [-3.72; 0.69] |
|  | Two | -1.61 | 0.23 | [-4.23; 1] |
|  | Three or more | -1.86 | 0.17 | [-4.49; 0.77] |
| **Income** *[Ref = Under 1,700 €]* | Between 1.700 € and 2.300 € | 0.41 | 0.76 | [-2.23; 3.05] |
|  | Between 2.300 € and 3.200 € | 1.04 | 0.47 | [-1.77; 3.85] |
|  | 3.200 € and more | 2.80 | **0.07** | [-0.25; 5.84] |
|  | No information | 1.99 | 0.28 | [-1.64; 5.63] |
| **Type of injury** *[Ref = 0]* | Injury type 1 | -1.48 | 0.49 | [-5.7; 2.74] |
|  | Injury type 2 | -4.54 | 0.39 | [-14.82; 5.73] |
|  | Injury type 3 | -1.05 | 0.63 | [-5.25; 3.16] |
|  | Injury type 4 | -2.16 | 0.13 | [-4.95; 0.64] |
|  | Injury type 5 | -0.59 | 0.68 | [-3.37; 2.19] |
|  | Injury type 6 | 0.32 | 0.88 | [-3.99; 4.63] |
|  | Injury type 7 | -0.14 | 0.92 | [-3.03; 2.75] |
|  | Injury type 8 | -4.91 | **0.04** | [-9.52; -0.3] |
| **Complication in healing process** *[Ref = Not present]* | Complication present | 0.45 | 0.85 | [-4.29; 5.19] |
| **Subjective prognosis on return to work** *[Ref = Unlikely]* | Not sure | -0.25 | 0.88 | [-3.54; 3.05] |
|  | Sure | 4.01 | **0.02** | [0.68; 7.35] |
| **Satisfaction with remedy supply** *[Ref = Dissatisfied to moderately satisfied]* | Satisfied | 0.73 | 0.63 | [-2.21; 3.66] |
|  | Strongly satisfied | 2.67 | **0.09** | [-0.44; 5.78] |
| **Injury localization** *[Ref = One location]* | Two | -1.79 | 0.21 | [-4.61; 1.02] |
|  | Three or more | 2.65 | 0.21 | [-1.5; 6.8] |
| **Severity of injury** *[Ref = 1V]* | >2S | 1.39 | 0.59 | [-3.7; 6.48] |
|  | >2V | 0.56 | 0.85 | [-5.23; 6.35] |
|  | 1S | -1.67 | 0.15 | [-3.94; 0.6] |
|  | 2S | -1.35 | 0.47 | [-5.01; 2.31] |
|  | 2V | 2.76 | 0.13 | [-0.81; 6.33] |
| **Treatment type pre-admission** *[Ref = Not this treatment]* | Physiotherapy | 0.79 | 0.61 | [-2.3; 3.89] |
|  | Occupational therapy | -1.11 | 0.37 | [-3.54; 1.33] |
|  | Massage/lymphatic drainage | -0.19 | 0.85 | [-2.08; 1.7] |
|  | Medical training therapy | -2.91 | **0.05** | [-5.76; -0.06] |
|  | Psychological therapy | 0.94 | 0.62 | [-2.74; 4.62] |
|  | Other | 1.47 | 0.38 | [-1.84; 4.77] |
|  | None | 1.25 | 0.51 | [-2.49; 4.98] |
| **Pain under stress** |  | -0.07 | **0.00** | [-0.12; -0.03] |
| **Pain at rest** |  | -0.04 | **0.08** | [-0.1; 0.01] |
| **Number of days between acute clinic and inpatient rehabilitation** |  | -0.01 | 0.72 | [-0.04; 0.03] |
| **Smoking status** *[Ref = Not smoking]* | Smoking | -1.36 | 0.19 | [-3.41; 0.69] |
| **Heavy drinking** *[Ref = No risk]* | Increased risk | 2.14 | **0.09** | [-0.31; 4.58] |
|  | Risk | 0.88 | 0.47 | [-1.52; 3.29] |
|  | No information | -2.05 | 0.24 | [-5.46; 1.35] |
| **Visible consequences of the accident are burdening (e.g., scars)** *[Ref = Does not apply]* | Partially applies | -2.20 | **0.05** | [-4.41; 0.02] |
|  | Applies | -1.88 | 0.15 | [-4.46; 0.7] |
| **Sleep** |  | -0.05 | **0.01** | [-0.08; -0.01] |
| **Regular engagement in sports before the accident** *[Ref = No sport]* | Below the recommended level | -0.17 | 0.89 | [-2.74; 2.39] |
|  | According to recommended level | 0.17 | 0.87 | [-1.93; 2.28] |
| **Working sector (WS***) [Ref = Not this sector]* | WS 1 | -10.43 | 0.33 | [-31.31; 10.45] |
|  | WS 2 | -10.62 | 0.32 | [-31.38; 10.13] |
|  | WS 3 | -11.31 | 0.29 | [-32.07; 9.45] |
|  | WS 4 | -8.93 | 0.43 | [-30.96; 13.11] |
|  | WS 5 | -9.77 | 0.36 | [-30.55; 11] |
|  | WS 6 | -10.34 | 0.33 | [-31.21; 10.54] |
|  | WS 7 | -10.32 | 0.34 | [-31.76; 11.11] |
|  | WS 8 | -9.89 | 0.35 | [-30.74; 10.95] |
|  | WS 9 | -6.55 | 0.55 | [-28.13; 15.03] |
| **General: Looking after one’s health** *[Ref = Slightly]* | Moderately | 1.62 | 0.30 | [-1.42; 4.66] |
|  | Strongly | 2.00 | 0.23 | [-1.29; 5.28] |
| **Socializing: to which extent did physical health or emotional problems affect social contacts in the last 4 weeks** *[Ref = Not at all]* | Slightly | 0.93 | 0.37 | [-1.12; 2.99] |
|  | Strongly | -0.39 | 0.77 | [-3.01; 2.23] |
| **Possibility of pursuing again hobby** *[Ref = No]* | Yes | 0.81 | 0.44 | [-1.24; 2.87] |
| **Feeling of support by family and friends** *[Ref = Not supported to moderately supported]* | Supported | -1.72 | 0.28 | [-4.83; 1.39] |
|  | Strongly supported | -2.24 | 0.18 | [-5.48; 1] |
| **Feeling of support by company/colleagues** *[Ref = Not supported to moderately supported]* | Supported | 1.56 | 0.16 | [-0.61; 3.73] |
|  | Strongly supported | 0.89 | 0.47 | [-1.53; 3.3] |
| **School education** *[Ref = Graduation up to grade 12]* | No graduation | 3.38 | 0.21 | [-1.89; 8.66] |
|  | Graduation up to grade 9 | -1.03 | 0.41 | [-3.47; 1.41] |
|  | Graduation up to grade 10 | -0.68 | 0.56 | [-2.95; 1.6] |
| **Acceptance of the new situation by family** *[Ref = Not accepted to moderately accepted]* | Having no family | 1.66 | 0.51 | [-3.27; 6.59] |
|  | Accepted | 2.68 | 0.17 | [-1.16; 6.52] |
|  | Strongly accepted | 2.79 | 0.14 | [-0.93; 6.5] |
|  | No information | 4.14 | 0.10 | [-0.81; 9.09] |
| **Self-efficacy** |  | -1.66 | **0.04** | [-3.25; -0.07] |
| **Duration of inpatient rehabilitation** |  | -0.01 | 0.70 | [-0.03; 0.02] |
| **Signs of depression: PHQ-4** *[Ref = No]* | Yes | -0.30 | 0.80 | [-2.58; 1.98] |
| **Signs of anxiety: PHQ-4** *[Ref = No]* | Yes | -2.69 | **0.06** | [-5.45; 0.07] |
| **EQ-VAS at baseline (t1)** |  | 0.31 | **0.00** | [0.26; 0.36] |
| **WHODAS-Score** |  | -0.05 | 0.10 | [-0.1; 0.01] |
| **Ongoing legal disputes related to accident** *[Ref = No]* | Yes | -0.72 | 0.51 | [-2.89; 1.45] |
| **Stressful life event in the last 12 months** *[Ref = No]* | Yes | 2.88 | **0.00** | [0.89; 4.87] |
| **Current financial concerns** *[Ref = None]* | Yes | 0.68 | 0.59 | [-1.8; 3.16] |
|  | No information | -1.91 | 0.17 | [-4.6; 0.79] |
| **Accessible housing** *[Ref = Not accessible]* | Accessible | -0.35 | 0.70 | [-2.11; 1.42] |
| **Living situation** *[Ref = Alone]* | With others | 2.15 | **0.09** | [-0.31; 4.6] |
| **Children** *[Ref = None]* | One or more | -0.25 | 0.78 | [-2.05; 1.55] |
| **Time from end of acute treatment to onset of post-acute treatment** |  | -0.02 | 0.45 | [-0.07; 0.03] |
| **Exposure to special environmental conditions (e.g., noise, drafts, cold, moisture) at workplace** *[Ref = No]* | Yes | -0.71 | 0.48 | [-2.7; 1.28] |
| **Case management: Availability of case management / coordination** *[Ref = No]* | Yes | -0.37 | 0.67 | [-2.1; 1.35] |
| **Demand for pension claim** *[Ref = No]* | Yes | -0.42 | 0.89 | [-6.34; 5.5] |
| **Language: Cultural background** *[Ref = Other]* | German | 0.48 | 0.79 | [-3.05; 4.01] |
| **Appraisal of the consequences of the accident** *[Ref = Somewhat to not at all burdensome]* | Quite burdensome | -0.63 | 0.55 | [-2.69; 1.44] |
|  | Extremely burdensome | -1.21 | 0.41 | [-4.10; 1.68] |
| **Current work status** *[Ref = Self-employed]* | Dependent employed | 0.23 | 0.89 | [-3.10; 3.56] |
| **Social burden** *[Ref =No]* | Yes | 1.89 | 0.12 | [-0.52; 4.31] |
| **Main earner** *[Ref =No]* | Yes | 0.43 | 0.72 | [-1.94; 2.8] |
|  | No information | -1.04 | 0.47 | [-3.83; 1.76] |
| **Disease gain** |  | 0.13 | 0.40 | [-0.17; 0.42] |
| **Rehab plan** *[Ref = No]* | Yes | 0.66 | 0.47 | [-1.12; 2.43] |
| **Resilience** |  | -0.02 | 0.62 | [-0.10; 0.06] |
| **AIC** 29,369.4 | | | | |
| **Interpretation example:** **1.** Subjective prognosis on return to work: An estimate of 4.01 for *Sure* *(Ref. = Unlikely)* indicates that patients who were confident about returning to work at baseline reported, on average, EQ-VAS scores 4.01 points higher over time than those who considered a return unlikely, controlling for all other variables. **2.** Severity of injury: The estimate of 2.76 for the *injury severity category “2V” (reference: 1V)* indicates that, on average, patients with this injury profile reported EQ-VAS scores 2.76 points higher over time than those with single severe injuries, adjusting for all other variables in the model.  **Bold values indicate p-values < 0.1**; 95% - CI: 95 % confidence interval  AIC: Akaike information criterion  Explanations: t2: discharge from inpatient rehabilitation, t3: 12 weeks, t4: 26 weeks, t5: 52 weeks, t6: 78 weeks after discharge  **Injury types:** Injury Type 1: Extensive or deep injuries of the skin and soft tissue mantle; amputation injuries; muscle compression syndromes (compartment syndromes); thermal or chemical damage; Injury Type 2: Injuries to the great vessels; Injury Type 3: Severe chest or abdominal injuries with organ involvement including kidneys or urinary tract; Injury Type 4: Complex fractures of the large tubular bones, especially multiple or open fractures; Injury Type 5: Severe injuries to large joints; Injury Type 6: Severe injuries to the hand; Injury Type 7: Complex fractures of the facial skull and torso skeleton; Injury Type 8: Multiple injuries with severe manifestations  **Severity of injury:** 1V = one diagnosis: no severe injury, 1S = one diagnosis: severe (S) injury, 2V = two diagnoses: no severe injury, 2S = two diagnoses: at least one severe injury, >2V = 2 diagnoses: no severe injury, >2S = 2 diagnoses at least one severe injury  **Pain at rest / Pain under stress:** 0 = no pain to 100 = excruciating pain  **Sleep**: 0 = very good to 100 = very bad  **PHQ-4:** Patient Health Questionnaire  **WHODAS:** WHO Disability Assessment Schedule, 0 = no disability to 100 = complete disability  **Working sector (WS):** WS 1: Agriculture, forestry, animal husbandry and horticulture, WS 2: Raw material extraction, production and manufacturing, WS 3: Construction, architecture, surveying and building services engineering, WS 4: Natural sciences, geography and IT, WS 5: Transportation, logistics, protection and security, WS 6: Commercial services, trade in goods, distribution, hotel and tourism, WS 7: Business organization, accounting, law and administration, WS 8: Health, social services, teaching and education, WS 9: Linguistics, literature, humanities, social and economic sciences, media, art, culture and design | | | | |

**Supplementary Table 5:** Overview of variables, questions, response options and recoding

| **Variables** | **Questions / Descriptions** | **Instruments / Source** | **Response options** | **Recoding** |
| --- | --- | --- | --- | --- |
| **Health Problem** | | | | |
| **BMI** | Body Mass Index: weight in kg / height in m^2 | -- | weight [kg]  height [m] |  |
| **EQ-VAS: Current state of health** | Your health today. | EuroQol five dimensions five levels (EQ-5D-5L), visual analog scale (EQ-VAS) (1) | score [0 = worst health you can imagine to 100 = best health you can imagine] | -- |
| **EQ-5D Index** | General health-related quality of life | EuroQol five dimensions five levels (EQ-5D-5L) (1), Index: combination of the five levels | score [- 0.205 = worst health you can imagine to 1 = best health you can imagine] | -- |
| **Comorbidities: Pre-existing conditions** (Ref = 0) | 14 Items (e.g. cardiovascular disease) | Work Ability Index (WAI) short form (2), Item 3 | 2 = yes, own opinion  1 = yes, physician’s diagnosis  0 = no | 0 = no physician’s diagnosis  1 = one physician’s diagnosis  2 = two physician’s diagnoses  3 = three or more physician’s diagnoses |
| **Type of injury** |  |  |  |  |
| - Injury Type 1: Extensive or deep injuries of the skin and soft tissue mantle; amputation injuries; muscle compression syndromes (compartment syndromes); thermal or chemical damage - Injury Type 2: Injuries to the great vessels - Injury Type 3: Severe chest or abdominal injuries with organ involvement including kidneys or urinary tract - Injury Type 4: Complex fractures of the large tubular bones, especially multiple or open fractures - Injury Type 5: Severe injuries to large joints - Injury Type 6: Severe injuries to the hand - Injury Type 7: Complex fractures of the facial skull and torso skeleton - Injury Type 8: Multiple injuries with severe manifestations | VAV-Numbers | Index of Injuries (VAV) of DGUV and SVLFG (3) | 0 = injury not present  1 = injury present | -- |
| **Severity of injury**  (Ref = 1V) | VAV-Numbers | Index of Injuries (VAV) of DGUV and SVLFG (3) | 1V = one diagnosis: no severe injury  1S = one diagnosis: severe (S) injury  2V = two diagnoses: no severe injury  2S = two diagnoses: at least one severe (S) injury  >2V = > 2 diagnoses: no severe injury  >2S = > 2 diagnoses at least one severe (S) injury | -- |
| **Injury localization** (Ref = 1) | Localization of injury |  | 1 = Head (without facial skull)  2 = Facial skull / face  3 = Neck (spine)  4 = Thorax  5 = Abdomen  6 = Back/spine (thoracic or lumbar spine)  7 = Upper extremity (including shoulder)  8 =Lower extremity (including hip and pelvic bones) | 1 = One localization  2 = Two localization  3 = Three or more localization |
| **Complication in healing process**  (Ref = 0) | VAV-Number 11 | Index of Injuries (VAV) of DGUV and SVLFG (3) | 0 = complication not present  1 = complication present | -- |
| **Visible consequences of the accident are burdening (e.g., scars)** (Ref = 0) | The visible consequences of the accident, such as scars, burden me. |  | 1 = Applies  2 = Mostly applies  3 = Partially applies  4 = Barely applies  5 = Does not apply | 0 = Does not apply (4, 5)  1 = Partially applies (3)  2 = Applies (1, 2) |
| **Addictive behavior** |  |  |  |  |
| - Smoking Status   (Ref = 0) | Do you smoke? | German Health Update (GEDA, RKI) (4), Question 112 | 1 = yes, daily  2 = yes, occasionally  3 = no, not any more  4 = never smoked | 0 = non-smoker (3, 4)  1 = smoker (1, 2) |
| - Heavy Drinking   (Ref = 0) | 3 items (e.g. How often do you drink alcoholic beverages?) | Alcohol Use Disorders Identification Test (AUDIT-C) (5) | according to AUDIT-C | 0 = no risk  1 = increased risk for alcohol-related disorder  2 = risky alcohol consumption  3 = no information |
| **Functioning** | | | | |
| **Emotional functions:**   - **Signs of anxiety: PHQ-4** - **Signs of depression: PHQ-4** | 4 items (Over the last two weeks, how often have you been bothered by the following problems? E.g. feeling nervous, anxious or on edge) | Patient Health Questionnaire (PHQ-4) (6) | 0 = not at all  1 = several days  2 = more than half the days  3 = nearly every day | 0 = no sign  1 = signs of depression/anxiety |
| **Sensation of pain** |  |  |  |  |
| - Pain at rest | How severe is your pain at rest? | Visual analog scale | [0 = no pain to 100 = excruciating pain] | -- |
| - Pain under stress | How severe is your pain under stress? | Visual analog scale | [0 = no pain to 100 = excruciating pain] | -- |
| - Sleep | How well do you sleep? | Visual analog scale | [0 = very good to 100 = very bad] | -- |
| **Looking after one’s health**  (Ref = 0) | How much do you care about your health in general? | German Health Update (GEDA, RKI) (4), Question 134 | 1 = very much  2 = much  3 = moderate  4 = less  5 = not at all | 0 = less / not at all (4, 5)  1 = moderate (3)  2 = very much / much (1, 2) |
| **WHODAS: Limitations and restrictions in activities and participation (disability)** | 12 Items (In the past 30 days, how much difficulty did you have in…: e.g. Taking care of your household responsibilities?) | WHO Disability Assessment Schedule 2.0 (WHODAS 2.0) (7) | 1 = non  2 = mild  3 = moderate  4 = severe  5 = extreme / cannot do | score [0 = no disability to 100 = complete disability] |
| **Regular engagement in sports before the accident** | Did you regularly engage in sports before the accident? (Multiple answers are possible here) | icfPROreha project group | 0 = No, no regular sports  1 = Yes, moderate endurance-oriented activity (e.g., brisk walking) for less than 150 minutes per week  2 = Yes, moderate endurance-oriented activity (e.g., brisk walking) for at least 150 minutes per week  3 = Yes, intense endurance-oriented activity (e.g., fast cycling or running) for less than 75 minutes per week  4 = Yes, intense endurance-oriented activity (e.g., fast cycling or running) for at least 75 minutes per week  5 = Yes, muscle-strengthening physical activities (e.g., exercises) on less than 2 days per week  6 = Yes, muscle-strengthening physical activities (e.g., exercises) on at least 2 days per week | 0 = No sports (0)  1 = Below the recommended level (1, 3, 5)  2 = According to recommended level (2, 4, 6) |
| **Possibility of pursuing again hobby** (Ref = 0) | Do you think you will be able to pursue your hobby again in a year? | icfPROreha project group | 0 = no  1 = yes | -- |
| **Socializing** | To what extent have your physical health or mental health issues affected your usual interactions with family members, friends, neighbors, or acquaintances over the past four weeks? | icfPROreha project group | 1 = Not at all  2 = Somewhat  3 = Moderately  4 = Quite  5 = Stronlgy | 0 = Not at all (1)  1 = Slightly (2, 3)  2 = Strongly (4, 5) |
| **Environmental Factors** | | | | |
| **Type of accident - work or leisure accident** | Please answer some questions about your accident: I had a ... | icfPROreha project group | 1 = work accident  2 = commuting accident  3 = Leisure/sports accident  4 = Traffic accident  5 = Home/garden accident | 1 = work accident (1, 2)  2 = no work accident (3-5) |
| **Accessible housing** | What is your housing situation? | icfPROreha project group | 1 = My apartment is accessible at ground level  2 = My apartment is located on a floor/story  3 = My apartment is accessible by elevator  4 = My apartment is accessible without an elevator" | 0 = not accessible (2, 4)  1 = accessible (1, 3) |
| **Professional sector** |  |  |  |  |
| - Sector 1: Agriculture, forestry, animal husbandry and horticulture - Sector 2: Raw material extraction, production and manufacturing - Sector 3: Construction, architecture, surveying and building services engineering - Sector 4: Natural science, geography and information technology - Sector 5: Transport, logistics, protection and security - Sector 6: Commercial services, goods trade, distribution, hotel and tourism - Sector 7: Business organization, accounting, law and administration - Sector 8: Health, social services, teaching and education - Sector 9: Language, literature, humanities, social and economic sciences, media, arts, culture and design - Sector 10: Military   (Ref = 0) | In which professional sector do you work? | German Classification of Occupations 2010 (8) | 0 = no  1 = yes | -- |
| **Ongoing legal disputes related to the accident** (Ref = 0) | Are legal disputes still on-going in connection with the accident? | icfPROreha project group | 0 = no  1 = yes | -- |
| **Treatment: time from end of acute treatment to onset of post-acute treatment** | How many days were there between your discharge from the acute hospital and the start of rehabilitative measures (e.g. physiotherapy)? | icfPROreha project group | [days] | -- |
| **Treatment: Number of days between acute clinic and inpatient rehabilitation** | Calculation: Difference between admission to rehabilitation and discharge of acute clinic | -- | [days] | -- |
| **Treatment: Duration of inpatient rehabilitation** | Calculation | -- | [days] | -- |
| **Treatment: Treatment type pre-admission**   - Physiotherapy - Occupational therapy - Massage / Lymphatic drainage - Medical training therapy - Psychological counseling / therapy - Other treatment - None   (Ref = 0) | What treatments / interventions did you receive after discharge from the acute care hospital? | icfPROreha project group | 0 = no  1 = yes | -- |
| **Case management: Availability of case management / coordination**  (Ref = 0) | Do you already have personal contact with an occupational aide, rehab manager, or insurance case manager? | icfPROreha project group | 0 = no  1 = yes | -- |
| **Rehabilitation plan** (Ref = 0) | Has a goal agreement / rehab plan been completed? | icfPROreha project group | 0 = no  1 = yes | -- |
| **Current financial concerns**  (Ref = 0) | Do you currently have financial difficulties? | icfPROreha project group | 0 = no  1 = yes  2 = no information | -- |
| **Feeling of support by family and friends** (Ref = 0) | How much do you feel supported by your family and friends? | icfPROreha project group | 1 = not at all  2 = somewhat  3 = moderately  4 = quite  5 = very | 0 = Not supported to moderately supported (1, 2, 3)  1 = Supported (4)  2 = Strongly supported (5) |
| **Feeling of support by company/colleagues** (Ref = 0) | How much do you feel supported by your company and/or colleagues? | icfPROreha project group | 1 = not at all  2 = somewhat  3 = moderately  4 = quite  5 = very | 0 = Not supported to moderately supported (1, 2, 3)  1 = Supported (4)  2 = Strongly supported (5) |
| **Acceptance of the new situation by family** (Ref = 1) | To what extent does your family/partner accept the new situation resulting from the injury? | icfPROreha project group | 0 = I have no family/partner  1 = Not at all  2 = Somewhat  3 =Moderately  4 = Quite  5 = Very  6 = No information | 0 = No family/partner  1 = Not accepted to moderately accepted (1, 2, 3)  2 = Accepted (4)  3 = Strongly accepted (5)  4 = No information (6) |
| **Stressful life events in the last 12 months** (Ref = 0) | Has there been at least one stressful life event (e.g. death of spouse/partner/family member, divorce or separation from spouse/partner, illness of a family member, dismissal without notice, unemployment, change in financial situation) in the last 12 months? | icfPROreha project group | 0 = no  1 = yes | -- |
| **Satisfaction with remedy supply** | How satisfied are you with the current provision of therapeutic treatments, i.e., with the services such as physical therapy, exercise treatments, occupational therapy, etc.? | icfPROreha project group | 1 = Very satisfied  2 = Satisfied  3 = Moderately satisfied  4 = Dissatisfied  5 = Very dissatisfied | 0 = Dissatisfied to moderately satisfied (3, 4, 5)  1 = Satisfied (2)  2 = Strongly satisfied (1) |
| **Personal Factors** | | | | |
| **Age at admission** |  | --- | [in years] | -- |
| **Gender** (Ref = female) |  | --- | 0 = female  1 = male | -- |
| **Family situation** |  | icfPROreha project group |  |  |
| - Living situation (Ref = 0) | Do you live… |  | 0 = alone  1 = with others | -- |
| - Children (Ref = 0) | How many financially dependent children do you have? |  | [number] | 0 = no children  1 = children |
| - Social burden (Ref = 0) | Are there any special circumstances in your family that burden you? |  | 0 = no  1 = yes, care for a relative  2 = yes, single parent  3 = yes, other | 0 = no social burden  1 = social burden (1, 2, 3) |
| **Education** (Ref = 3) | What is your highest general school leaving certificate? | German General Social Survey (ALLBUS) 2016 (9), question F056 | 1 = pupil  2 = no graduation  3 = 8th or 9th grade  4 = 10th grade  5 = entrance qualification for a university of applied sciences  6 = higher education entrance qualification  7 = other graduation / free text | 0 = no graduation (2)  1 = graduation up to grade 9 (3)  2 = graduation up to grade 10 (4)  3 = graduation up to grade 12 (5, 6)  (free text (7) was allocated to other categories; pupil was omitted) |
| **Language: Cultural background** | What is/are your native language(s)? | icfPROreha project group | 1 = German  2 = Turkish  3 = Polish  4 = Russian  5 = Italian  6 = other | 0 = German  1 = other |
| **Social status** |  |  |  |  |
| - Income (Ref = 1) | What is the monthly net income of your household? [€] | According to: German General Social Survey (ALLBUS) 2016 (9), question F115 | 1 = < 900  2 = 900 to < 1300  3 = 1300 to < 1700  4 = 1700 to < 2300  5 = 2300 to <3200  6 = 3200 to < 4000  7 = 4000 to < 5000  8 = ≥ 5000  9 = no information | 1 = below 1700 (1-3)  2 = 1700 to < 2300 (4)  3 = 2300 to <3200 (5)  4 = 3200 or more (6-8)  5 = no information (9) |
| - Main earner (Ref = 0) | The household income corresponds to my personal income | icfPROreha project group | 0 = no  1 = yes  2 = no information | -- |
| **Employment status** |  |  |  |  |
| - Work: Situation pre-accident   (Ref = 0) | What was your professional situation before the accident? | icfPROreha project group | 1 = fully employed  2 = partly employed (half-day, hourly)  3 = unable to work  4 = registered jobseeker  5 = other | 0 = not working (3, 4);  1 = partly employed (2);  2 = fully employed (1);  "other" was assigned, if possible; |
| - Work status: Employment type   (Ref = 0) | In your current job, are you...? | icfPROreha project group | 1 = …self-employed (own business, own office or similar)  2 = … dependent employed (working as worker, employee or civil servant) | -- |
| **Exposure to special environmental conditions (e.g., noise, drafts, cold, moisture) at workplace** (Ref = 0) | Are you frequently exposed to special environmental conditions (e.g., noise, drafts, cold, moisture) at your workplace? | icfPROreha project group | 0 = no  1 = yes | -- |
| **Subjective prognosis on return to work**  **(**Ref = 0) | Do you think that, based on your current state of health, you will still be able to do your current job in a year's time? | icfPROreha project group | 0 = unlikely  1 = not sure  2 = very likely | -- |
| **Demand for pension claim**  (Ref = 0) | I think I will probably apply for / get a pension in the near future. | Screening Instrument for the Access to Work-Related Multimodal Rehabilitation (SIMBO) (10), Item SI 6, response option 5 | 0 = no  1 = yes | -- |
| **Self-efficacy** | 3 items (e.g. I can rely on my abilities in difficult situations) | Short Scale for Measuring General Self-efficacy Beliefs (ASKU) (11) | 1 = disagree strongly to 5 = agree strongly | score [1 to 5; higher scores indicate a more pronounced factor] |
| **Consequences accident: Appraisal of the consequences of the accident**  (Ref = 0) | How do you rate the consequences of the accident? | icfPROreha project group | 1 = not at all burdensome  2 = little burdensome  3 = somewhat burdensome  4 = quite burdensome  5 = extremely burdensome | 0 = somewhat to not at all burdensome (1,2,3)  1 = quite burdensome (4)  2 = extremely burdensome (5) |
| **Resilience** | 13 items (e.g. I like myself.) | Resilience Scale (RS-13) (12) | 1 = disagree to 7 = agree | sum score (13-91)  13-66 = low  67-72 = moderate  73-91 = high |
| **Disease gain** | 4 items (e.g. When I'm feeling bad, someone is more likely to take care of me than usual.) | Patient Questionnaire for Assessment of Rehabilitation Motivation (PAREMO-20) (13), Items 2, 7, 14, 19 | 1 = not true  2 = rather not true  3 = rather true  4 = true | -- |

## Supplementary Figures

**Supplementary Figure 1** Flow chart of included and excluded patients and observations

**Supplementary Figure 2** Proportions: Working sector (WS)

**WS 1:** Agriculture, forestry, animal husbandry and horticulture, **WS 2:** Raw material extraction, production and manufacturing, **WS 3:** Construction, architecture, surveying and building services engineering, **WS 4:** Natural sciences, geography and IT, **WS 5:** Transportation, logistics, protection and security, **WS 6:** Commercial services, trade in goods, distribution, hotel and tourism, **WS 7:** Business organization, accounting, law and administration, **WS 8:** Health, social services, teaching and education, **WS 9:** Linguistics, literature, humanities, social and economic sciences, media, art, culture and design, **WS 10:** Military (8)

## Supplementary References

1. The-EuroQol-Group. EuroQol - a new facility for the measurement of health-related quality of life. Health Policy. 1990;16(3):199-208.

2. Ilmarinen J. The Work Ability Index (WAI). Occup Med. 2007;57(2):160.

3. German Social Accident Insurance and Social Insurance for Agriculture, Forestry and Horticulture. Injury Type Index: Injury Type Index with Explanations Including the Procedure for Severely Injured Types (Revised Version 2.0, as of July 1, 2018) 2018 [Available from: https://www.dguv.de/medien/landesverbaende/de/med_reha/documents/verletz3.pdf]

4. RKI. Questionnaire for the Study "Health in Germany Current" GEDA 2014/2015-EHIS. Journal of Health Monitoring. 2017;2(1):105-35.

5. Bush K, Kivlahan DR, McDonell MB, Fihn SD, Bradley KA, Project ftACQI. The AUDIT Alcohol Consumption Questions (AUDIT-C): An Effective Brief Screening Test for Problem Drinking. Archives of Internal Medicine. 1998;158(16):1789-95.

6. Kroenke K, Spitzer RL, Williams JB, Löwe B. An ultra-brief screening scale for anxiety and depression: the PHQ-4. Psychosomatics. 2009;50(6):613-21.

7. Ustun TB, Kostanjesek N, Chatterji S, Rehm J, WHO. Measuring health and disability : manual for WHO Disability Assessment Schedule (WHODAS 2.0) / edited by T.B. Üstün, N. Kostanjsek, S. Chatterji, J.Rehm. Geneva: World Health Organization; 2010.

8. Classification of Occupations (KldB) 2010: Federal Employment Agency (BfA) - Statistics; 2022 [Available from: https://statistik.arbeitsagentur.de/DE/Navigation/Grundlagen/Klassifikationen/Klassifikation-der-Berufe/KldB2010/KldB2010-Nav.html].

9. ALLBUS 2016: Questionnaire Documentation. GESIS – Leibniz Institute for the Social Sciences; 2016.

10. Streibelt M, Gerwinn H, Hansmeier T, Thren K, Müller-Fahrnow W. SIMBO: A Screening Instrument for Determining the Need for Medical-Vocational Oriented Measures in Medical Rehabilitation - Analyses of Construct Validity and Prognostic Accuracy. Rehabilitation. 2007;46:266-75.

11. Beierlein C, Kemper C, Kovaleva A, Rammstedt B. Short Scale for Measuring General Self-efficacy Beliefs (ASKU). 2013.

12. Leppert K, Koch B, Brähler E, Strauss B. The Resilience Scale (RS) - Examination of the Long Form RS-25 and a Short Form RS-13. Clinical Diagnostics and Evaluation. 2008;1:226-43.

13. Hafen K, Jastrebow J, Nübling R, Bengel J. Development of a Patient Questionnaire for Assessing Rehabilitation Motivation (PAREMO). Rehabilitation. 2001;40:3-11.
